# Activation of miR-34a impairs autophagic flux and promotes cochlear cell death via repressing *ATG9A*: implications for age-related hearing loss

**DOI:** 10.1038/cddis.2017.462

**Published:** 2017-10-05

**Authors:** Jiaqi Pang, Hao Xiong, Peiliang Lin, Lan Lai, Haidi Yang, Yimin Liu, Qiuhong Huang, Suijun Chen, Yongyi Ye, Yingfeng Sun, Yiqing Zheng

**Affiliations:** 1 Department of Otolaryngology, Sun Yat-sen Memorial Hospital, Sun Yat-sen University, Guangzhou, China; 2 Institute of Hearing and Speech-Language Science, Sun Yat-sen University, Guangzhou, China; 3 Guangdong Provincial Key Laboratory of Malignant Tumor Epigenetics and Gene Regulation, Medical Research Center, Sun Yat-sen Memorial Hospital, Sun Yat-sen University, Guangzhou, China; 4 Guangzhou Occupational Disease Prevention and Treatment Center, Guangzhou, China

## Abstract

Age-related hearing loss is a major unresolved public health problem. We have previously elucidated that the activation of cochlear miR-34a is correlated with age-related hearing loss in C57BL/6 mice. A growing body of evidence points that aberrant autophagy promotes cell death during the development of multiple age-related diseases. The aim of this study was to investigate the role of miR-34a-involved disorder of autophagy in the pathogenesis of age-related hearing loss. Our results showed that miR-34a expression was markedly upregulated in the aging cochlea accompanied with impairment of autophagic flux. In the inner ear HEI-OC1 cell line, miR-34a overexpression resulted in an accumulation of phagophores and impaired autophagosome–lysosome fusion, and led to cell death subsequently. Notably, autophagy-related protein 9A (*ATG9A*), an autophagy protein, was significantly decreased after miR-34a overexpression. Knockdown of *ATG9A* inhibited autophagy flux, which is similar to the effects of miR-34a overexpression. Moreover, ursodeoxycholic acid significantly rescued miR-34a-induced HEI-OC1 cell death by restoring autophagy activity. Collectively, these findings increase our understanding of the biological effects of miR-34a in the development of age-related hearing loss and highlight miR-34a as a promising therapeutic target for its treatment.

Age-related hearing loss (AHL), known as presbycusis, is a complex degenerative disease, afflicting ~50% of people by age 65 years and older.^1, 2^ Hearing loss can impair everyday communication in the elderly, causing loneliness and isolation^3^ that make AHL a risk factor for Alzheimer's disease, dementia, depression and other neuropsychiatric diseases.^3, 4, 5^ The detailed mechanisms underlying AHL remain largely unknown. AHL is a reflection of genetic predisposition as well as a lifetime of insults to the ear, such as the accumulation of noise exposure, ototoxic drugs and diseases.^6, 7^ Cochlear degeneration is a common feature in AHL both in humans and animal models,^8^ including hair cell loss, stria vascularis and spiral ganglion neurons degeneration.^3, 9^ The loss of cochlear hair cell is well documented and considered as one of the major causes for AHL.^10, 11, 12, 13^ Because hair cells do not regenerate in mammals, clarifying the mechanism of hair cell death is regarded as an important strategy for the treatment of AHL.

Autophagy, a lysosome degradation pathway by which cells capture and deliver damaged proteins and cellular organelles, plays an important homeostatic role in keeping the metabolic balance between synthesis and degradation in stressful environment.^14, 15^ Macroautophagy is one of the classical form and the most studied type of autophagy. Briefly, the processes consist two stages: the early stage includes the induction and formation of the phagophore, the formation of the autophagosome, which captures damaged cellular proteins and organelles; the late stage is autophagosome–lysosome fusion, which is responsible for degradation and recycling.^16, 17^ Recently, aberrant autophagy has been implicated in a variety of neurodegenerative disorders, cancers and autoimmune diseases.^17, 18, 19, 20^ Aberrant autophagy makes the clearance of the misfolding and aggregated proteins and organelles ineffectively, contributing to many neurodegenerative disorders, such as Alzheimer's disease, Parkinson's disease and Huntington's disease.^18, 19, 21, 22, 23^ However, the role of autophagy in hearing development, maintenance and multiple types of hearing loss remains to be elucidated. Previous research revealed autophagy attenuates cisplatin and noise-induced hearing loss,^24, 25, 26^ while increased autophagic stress may account for premature AHL in SAMP8 mice.^27^

MicroRNAs (miRNAs or miRs) are endogenous, small (20–24 nt), non-coding RNAs that post-transcriptionally regulate messenger RNA (mRNA) stability and ultimate translation. Growing studies proved that miRNAs regulate aging in worms (*Caenorhabditis elegans*), mice and humans.^28, 29^ Recently, miRNAs were observed in the cochlea and suggested to play an important role in the cochlear pathology.^11, 30^ Of interest is miR-34a, which has been implicated as a prime candidate inducing senescence, cell cycle arrest, autophagy and cell death.^31, 32^ Our previous study has confirmed that miR-34a is linked to AHL,^11^ but the detailed mechanism is not fully understood. Recently, miR-34a was reported to be pathologically altered and observed as one of the modulators that regulate autophagy in many neurodegenerative diseases.^23, 33^

Thus, we hypothesize that miR-34a activation causes cochlear cell death and AHL via modulating autophagy, and the strategies aimed at inhibiting miR-34a activity may be beneficial in treating AHL through autophagy restoration. In the present study, cochlear miR-34a and autophagy status were examined in C57BL/6 mice, a mouse model of AHL. Additionally, modulation of autophagy by miR-34a and the protective effect of ursodeoxycholic acid (UDCA), a suppressant of miR-34a, was assessed in HEI-OC1 cells.

## Results

### C57BL/6 mice develop hearing loss and cochlear hair cell loss with aging

**Figure 1:**
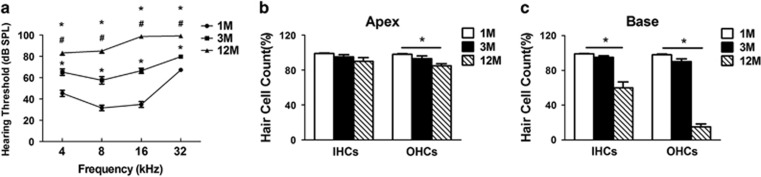
Hearing loss and cochlear hair cell loss in C57BL/6 mice with aging. (**a**) Elevated ABR thresholds were observed in C57BL/6 mice at 4, 8, 16 and 32 kHz according to the age. *P<0.05 compared with 1 month; ^#^*P*<0.05 compared with 3 months. *N*=20 per age group. Hair cell counts obtained from two representative cochlear locations, in the apical turn (**b**) and basal turn (**c**), at different ages. *P<0.05. *N*=6 in each group. Date were represented as the mean±S.D. 1M, 1 month old; 3M, 3 month old; 12M, 12 month old

Auditory brainstem response (ABR) measurement was used to monitor the progression of AHL. The average thresholds in 3-month old mice (65.3±3.0 dB at 4 kHz, 57.5±3.6 dB at 8 kHz, 66.5±2.5 dB at 16 kHz and 79.8±1.7 dB at 32 kHz) were significantly elevated compared with those in 1-month-old mice (45.3±2.8 dB at 4 kHz, 31.5±2.5 dB at 8 kHz, 34.8±2.8 dB at 16 kHz and 67.3±1.2 dB at 32 kHz). The average thresholds in 12-month-old mice (83.3±1.9 dB at 4 kHz, 85.0±2.0 dB at 8 kHz, 98.8±0.5 dB at 16 kHz and 99.3±0.4 dB at 32 kHz) (4 kHz: F_(2,57)_=52.7, *P*<0.001; 8 kHz: F_(2,57)_=92.4, *P*<0.001; 16 kHz: F_(2,57)_=221.4, *P*<0.001; 32 kHz: F_(2,57)_=168.3, *P*<0.001) were significantly elevated compared with those in 1- and 3-month-old mice, indicating that C57BL/6 mice developed early-onset AHL (Figure 1a). To determine whether the functional deficits corresponded to the extent and the localization of hair cell loss, hair cell counts were proceeded by cochleae surface preparations and calculation after the ABR measurements. There was no inner hair cell (IHC) or outer hair cell (OHC) loss in 1-month-old mice at the apical and basal cochlear turns. In 3-month-old mice, only a minor loss of OHCs was apparent in the basal region. Twelve-month-old animals showed <15% loss of OHCs (85.0±2.4%) and a 10% loss of IHCs (90.0±4.3%) in apical turn (OHCs: F_(2, 15)_=7.5, *P*=0.006; Figure 1b), while a 85% loss of OHCs (15.0±3.4%) and a 40% loss of IHCs (60.0±6.6%) in the basal turn (IHCs: F_(2, 15)_=29.1, *P*<0.001; OHCs: F_(2, 15)_=277.5, *P*<0.001; Figure 1c).

### MiR-34a is upregulated and autophagic flux is impaired in the cochlea of C57BL/6 mice with aging

**Figure 2:**
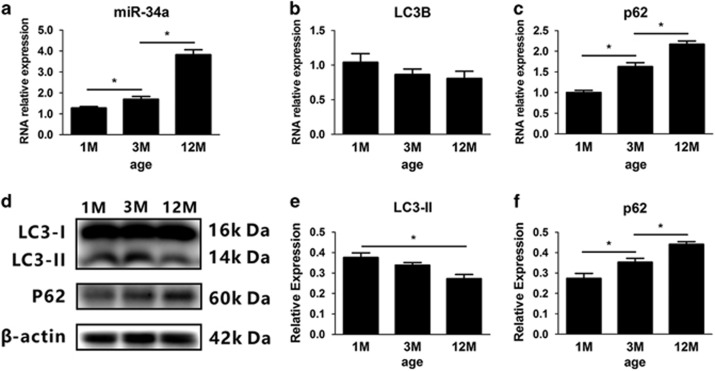
miR-34a activation and the impairment of autophagic flux in the cochlea with aging. (**a**–**c**) Quantitative real-time polymerase chain reaction analysis of miR-34a, *LC3B* and *p62* in different ages of C57BL/6 mice. Date were represented as the mean±S.E.M. obtained from four independent experiments. (**d**–**f**) Western blot and densitometry of *LC3-II* and *p62*. *P<0.05. Date were represented as the mean±S.D. obtained from four independent experiments. 1 M, 1 month old; 3 M, 3 month old; 12 M, 12 month old

We sought to determine the correlation of miR-34a and autophagy in the aging cochlea. In this sense, we also examine the mRNA expression of *LC3B* and *p62* that are correlated with autophagy. The elongation of the edges of the phagophore membrane contains a second ubiquitin-like protein, an *ATG8* family member, *LC3*, which is cleaved by *ATG4* to form cellular *LC3-I*. Then, cellular *LC3-I* is covalently conjugated to phosphatidylethanolamine on the phagophore membrane, in which it is known as *LC3-II*.^34^ Therefore, *LC3-II* is specifically associated with phagophore and autophagosome membranes serving as a widely used marker to monitor autophagy levels. Another autophagy marker is *p62*, which is efficiently degraded upon autophagy induction and serves as an index of autophagic degradation.^35^ The expression of miR-34a, *LC3B* and *p62* mRNA was examined in the different ages of C57BL/6 mice via real-time PCR. The miR-34a expression was significantly upregulated during aging (F_(2,10)_=83.802, *P*<0.001; Figure 2a). Conversely, *LC3B* mRNA expression was slightly decreased in the cochlea with aging (F_(2,10)_=1.378, *P*=0.30; Figure 2b), whereas *p62* mRNA expression showed a significant age-related elevation (F_(2,10)_=60.771, *P*<0.001; Figure 2c). Consistent with the findings in mRNA alteration, western blot analysis revealed similar changes in *LC3-II* and *p62* protein in different ages (Figure 2d). *LC3-II* protein levels in the cochlea were decreased (F_(2,10)_=30.769, *P*<0.001; Figure 2e), whereas *p62* expression was elevated with aging (F_(2,10)_=77.077, *P*<0.001; Figure 2f).

### MiR-34a overexpression impairs autophagic flux and induces HEI-OC1 cell death

**Figure 3:**
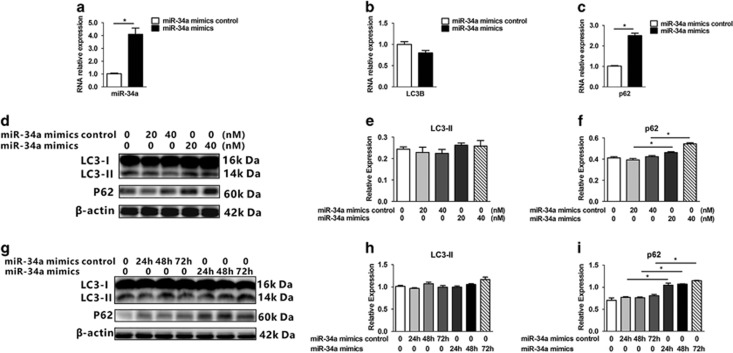
miR-34a modulates autophagy in HEI-OC1 cells. (**a**–**c**) Quantitative PCR (qPCR) analysis of miR-34a modulation of *LC3B*, *p62*. Date represent the mean±S.E.M. obtained from three independent experiments. (**d**) Western blot of *LC3-II* and *p62* under various treatment with a miR-34a mimic (20 and 40 nM) or miR-34a mimic control. (**e** and **f**) Densitometry analysis of (**d**). (**g**) Western blot of LC3-II and p62 under various time after miR-34a mimic transfection. (**h** and **i**) Densitometry analysis of (**g**). *P<0.05. Date were represented as the mean±S.D. obtained from three independent experiments

To validate the hypothesis that increased miR-34a is associated with autophagy impairment in aging cochlea, the HEI-OC1 cell lines, an extensively used cell line used to elucidate pathways of hair cell pathology,^36, 37^ were transfected with a miR-34a mimic or a negative control. Real-time PCR analysis showed that the miR-34a mimic increased *p62* mRNA levels without a significative change of *LC3B* expression (miR-34a: *t*_(4)_=−6.2, *P*=0.003 (Figure 3a); *LC3B*: *t*_(4)_=2.45, *P*=0.067 (Figure 3b); p62: *t*_(4)_=−12.8, *P*<0.001; Figure 3c). Western blot findings demonstrated that miR-34a overexpression increased *p62* protein in a dose-dependent manner (p62 expression in miR-34a mimics 20 nM: *t*(4)=−7.6, *P*=0.002; in 40 nM: *t*_(4)_=−14.1, *P*<0.001; Figures 3d–f) and a time-dependent manner (*p62* expression at 24h after miR-34a mimics transfection: *t*_(4)_=−10.2, *P*=0.001; at 48h: *t*_(4)_=−28.4, *P*<0.001; at 72h: *t*_(4)_=−17.6, *P*<0.001; Figures 3g–i). These findings suggested that miR-34a may impair autophagic flux.

**Figure 4:**
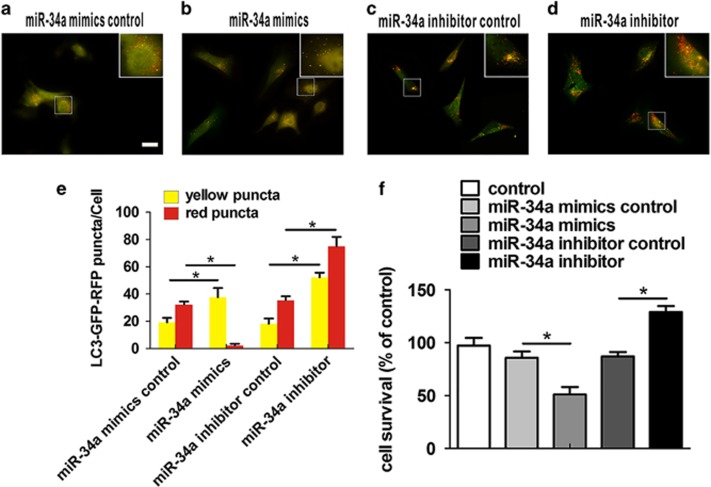
miR-34a promotes HEI-OC1 cells death by impairing autophagic flux. Fluorescence images of mRFP-GFP-*LC3* in HEI-OC1 cells treated with a miR-34a mimic control (**a**), miR-34a mimic (**b**), miR-34a inhibitor control (**c**) or miR-34a inhibitor (**d**). Scale bars: 10 *μ*m. Quantity analysis of yellow and red puncta was detected (**e**). Date were represented as the mean±S.E.M. obtained from five independent experiments. The MTS assay was performed to examine the viability of HEI-OC1 cells under various conditions (**f**). Date were represented as the mean±S.E.M. obtained from three independent experiments. *P<0.05

To determine whether miR-34a could inhibit actual autophagic flux in HEI-OC1 cells, mRFP-GFP-*LC3* adenoviral vectors were used to evaluate the autophagic level treated with miR-34a mimic or inhibitor. In general, *LC3* appears as a diffuse pattern in the cytoplasm. After autophagy is activated, *LC3* gathers and appears as a punctate pattern. The GFP signal is sensitive to the acidic conditions of the lysosome lumen, whereas mRFP is more stable. Therefore, the yellow punctum, which is colocalized of both GFP and mRFP fluorescence, indicates a compartment that has not fused with a lysosome, such as the phagophore or an autophagosome, whereas the red punctum from an mRFP signal without GFP corresponds to an amphisome or autolysosome. Thus, autophagic flux can be determined by evaluating the number of yellow and red puncta.^35^ Generally, both yellow and red puncta were in the cytoplasm (Figures 4a and c). As expected, HEI-OC1 cells treated with miR-34a mimic only showed accumulation of yellow puncta in the perinuclear region and cytoplasm (miR-34a mimics *versus* its control: yellow puncta: *t*(8)= 5.4, *P*=0.001; red puncta: *t*(8)= −30.3, *P*<0.001; Figures 4b and e), which implied miR-34a prevented the compartment from fusing with a lysosome and blocked autophagosome–lysosome fusion. Conversely, miR-34a inhibition increased both yellow and red puncta, showing the accelerated and unobstructed autophagic flux in HEI-OC1 cells (miR-34a inhibitor *versus* its control: yellow puncta: *t*(8)=14.4, *P*<0.001; red puncta: *t*(8)=11.3, *P*<0.001; Figures 4d and e). To explore the functional effect of miR-34a on cell survival, cell viability was measured in cells transfected with the miR-34a mimic and miR-34a inhibitor. Compared with the controls, the MTS (3-(4,5-dimethylthiazol-2-yl)-5-(3-carboxymethoxyphenyl)-2-(4-sulfophenyl)-2*H*-tetrazolium) assay showed that HEI-OC1 cells transfected with the miR-34a mimic had a reduced survival rate, whereas the miR-34a inhibitor promoted cell survival (miR-34a mimics *versus* its control: *t*_(4)_=6.4, *P*=0.003; miR-34a inhibitor *versus* its control: *t*_(4)_=−10.6*, P*<0.001; Figure 4f).

### MiR-34a regulates autophagy through *ATG9A* in HEI-OC1 cells

**Figure 5:**
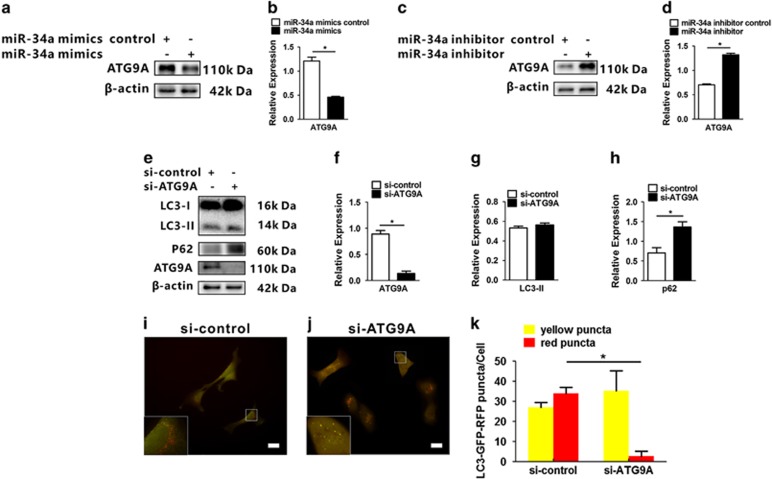
*ATG9A* is targeted by miR-34a in the regulation of autophagy. (**a** and **b**) HEI-OC1 cells transfected with miR-34a mimics and its control were subjected to western blot analysis for *ATG9A*. (**c** and **d**) Western blot and densitometry analysis performed after the transfection of miR-34a inhibitor and inhibitor control. (**e**–**h**) HEI-OC1 cells were transfected with si-control and si-*ATG9A*. Seventy-two hours post-transfection, total protein was harvested and subjected to western blot analysis for *ATG9A*, *LC3-II* and *p62*, and *β*-actin was used as a loading control. Quantification of band intensities normalized to *β*-actin and relative to control are shown below respective blots. Fluorescence images of mRFP-GFP-*LC3* in HEI-OC1 cells treated with a si-control (**i**) and si-ATG9A (**j**) with a nutrition-free medium for 6 h before fixation. Quantity analysis of yellow and red puncta was detected (**k**). Scale bars: 10 *μ*m. Date were represented as the mean±S.E.M. obtained from four independent experiments. *P<0.05

The previous study has demonstrated that *ATG9A* is one of the major targets of miR-34a^31^ and related to the growth of autophagic membranes.^35, 38^ To test whether miR-34a modulates autophagy via ATG9A in HEI-OC1, cells were transfected with a miR-34a mimic or miR-34a inhibitor, with a negative control miRNA mimic or inhibitor. Overexpression of miR-34a in HEI-OC1 cells resulted in a marked downregulation of *ATG9A* expression (*t*_(4)_=15.9, *P*<0.001; Figures 5a and b). Oppositely, inhibition of miR-34a increased *ATG9A* expression (*t*_(4)_=27.9, *P*<0.001; Figures 5c and d). Next, we sought to determine the effects of *ATG9A* depletion on autophagy using siRNA-mediated knockdown of *ATG9A* (Figure 5e). Knockdown of ATG9A resulted in an accumulation of *p62* without a significant change of LC3-II expression that was similar to that mediated by miR-34a overexpression (*LC3-II*: *t*_(4)_=−2.0, *P*=0.123; *p62*: *t*_(4)_=−6.1, *P*=0.004; Figures 5g and h). The Ad-mRFP-GFP-*LC3* transfection were performed before the si-*ATG9A* knockdown transfection. Interestingly, HEI-OC1 cells treated with si-*ATG9A* showed a few red puncta and plenty of yellow puncta in the perinuclear region and cytoplasm (yellow puncta: *t*_(4)_=−0.661, *P*=0.544; red puncta: *t*_(4)_=13.9, *P*<0.001; Figures 5i–k). Knockdown of *ATG9A* also led to the blockage of autophagosome–lysosome fusion as what miR-34a overexpression did, demonstrating the possibility that miR-34a inhibits autophagosome–lysosome fusion by deregulation of *ATG9A*.

### UDCA rescues HEI-OC1 cells from death through autophagy recovery

**Figure 6:**
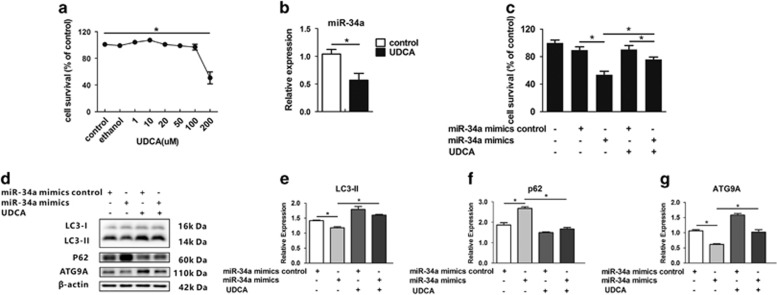
UDCA protects HEI-OC1 cells from miR-34a mimic-induced cell death. (**a**) Cell survival experiment was detected in various concentration of UDCA. (**b**) MiR-34a expression was performed after being treated with or without 10 *μ*M UDCA for 24 h. (**c**) The MTS assay was performed to examine the viability of HEI-OC1 cells under miR-34a overexpression with or without UDCA treatment. The HEI-OC1 cell viability assessed via MTS assay. (**d–g**) Western blot analysis and densitometry of *LC3-II*, *p62* and *ATG9A*. *P<0.05. Date were represented as the mean±S.E.M. obtained from three independent experiments

UDCA is a potent modulator of miRNA transcription involved in apoptosis, cell cycle control, proliferation and cell growth.^39^,^40^ As UDCA reduces wild-type miR-34a promoter activity after *p53* overexpression and hampers miR-34a expression by almost 40% in rat liver,^39, 41^ we wondered if UDCA treatment reduces miR-34a expression in HEI-OC1 cells. Cell survival experiment showed 10 *μ*M UDCA treatment in 24 h had the best effects of survival, and 200 *μ*M was median lethal dose (F_(7,16)_=53.7, *P*<0.001; Figure 6a). Ten micromolar UDCA treatment significantly decreased miR-34a expression (*t*_(4)_=5.5, *P*=0.005; Figure 6b). The MTS assay results showed that 10 *μ*M UDCA treatment significantly reduced miR-34a mimic-induced cell death (F_(4,10)_=41.1, *P*<0.001; Figure 6c). The western blot analysis indicated that 10 *μ*M UDCA treatment significantly restored autophagic flux impairment, by the increase of *p62* degradation and the recovery of *ATG9A* expression (*LC3-II*: F_(3,8)_=72.4, *P*<0.001; *p62*: F_(3,8)_=124.2, *P*<0.001; *ATG9A*: F_(3,8)_=161.0, *P*<0.001; Figures 6d–g).

## Discussion

**Figure 7:**
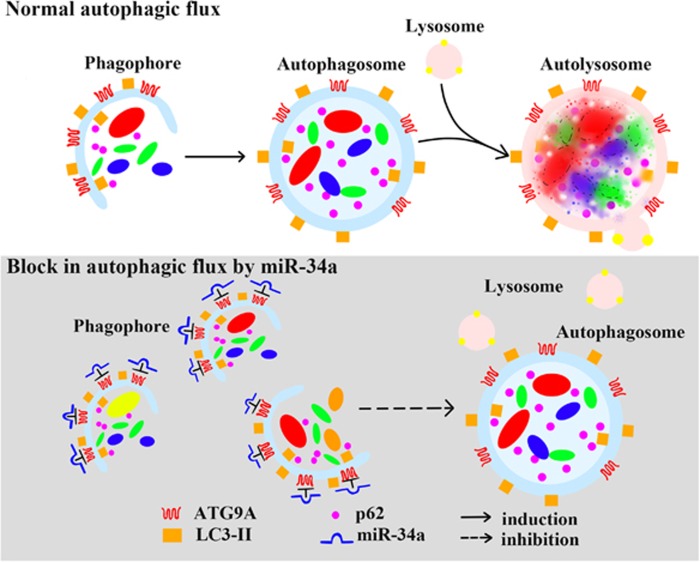
Schematic model demonstrating the elevation of miR-34a impairs autophagic flux through *ATG9A*. *ATG9A*, the only multipass transmembrane ATG protein, is required for the expansion of autophagic membranes. Under normal condition, the initiation of autophagy includes the formation of the phagophore, membrane closure to encapsulate contents in the autophagosome. Completion of the autophagosome is followed by fusion with lysosomes and degradation of the contents. We favor the hypothesis that in the case of miR-34a overexpression, miR-34a inhibition of *ATG9A* impairs autophagosome biogenesis leading to phagophore accumulation. Therefore, the formation of autophagosome is inhibited and the autophagic flux is limited. *LC3-II* is specifically associated with phagophore and autophagosome membranes serving as a widely used marker to monitor autophagy levels. Another autophagy marker is *p62*, which is efficiently degraded upon autophagy induction and serves as an index of autophagic degradation

In the present study, we demonstrated that miR-34a was activated in AHL accompanied with the impairment of autophagic flux. In addition, in HEI-OC1 cells, miR-34a inhibited autophagic flux through suppressing autophagy protein *ATG9A* (Figure 7). Moreover, UDCA treatment protected HEI-OC1 cells by inhibition of miR-34a and rescued autophagic flux.

AHL is a progressive neurodegenerative disorder in the auditory system.^1, 3^ One of the main causes of AHL is thought to be the irreversible loss of cochlear hair cells in the inner ear.^1, 3, 13^ However, the detailed mechanism underlying cochlear hair cell death in AHL remains largely unknown. Because the impairment of autophagic flux is suggested to play an important role in neurodegenerative diseases and aging, we were interested in determining whether aberrant autophagy is involved in cochlear hair cell death and AHL induction. In the present study, we found that miR-34a overexpression increased *p62* and impaired the autophagic flux *in vitro*, which is similar to other aging studies.^42, 43, 44^ Meanwhile, miR-34a promoted HEI-OC1 cells death, which is consistent with our previous study.^11^ We consider that the activation of miR-34a increased *p62* expression through the impairment of autophagic flux and promoted HEI-OC1 cells death. We also found that miR-34a increase was accompanied by the autophagy impairment in the aging cochlea of C57BL/6 mice. These findings suggested that miR-34a modulation of autophagy might be involved in the pathogenesis of AHL in C57 mice. Taken together, we have reason to believe that miR-34a-induced autophagy impairment is correlated with cochlear hair cell death and might contribute to AHL.

The mechanisms underlying miR-34a blockage of autophagy remains largely unknown. In *C. elegans*, miR-34a modulates lifespan via directly repressing the autophagy gene *ATG9A*.^31^ In mammalian models, gene *ATG9A* was proved as one of miR-34a target genes and its inhibition was found in neural stem cell differentiation^45^ and cardiac hypertrophy,^46^ while miR-34a was overexpressing. *ATG9A* protein is necessary for optimal autophagy.^47, 48, 49^ The current hypothesis is that *ATG9A* delivers lipids, which is required for the expansion of autophagosomes.^35, 38^ As the only transmembrane ATG protein, *ATG9* was supposed to associate with many other compartments, including recycling endosomes, early endosomes and late endosomes, and it may also be possible that *ATG9A* delivers regulators to the growing phagophore.^50^ In parkin-mediated mitophagy, the relocalization of transcription factor EB, a master regulator of lysosomal biogenesis, required ATG9A.^51^ Whether miR-34a blocked autophagic flux via *ATG9A* in HEI-OC1 cells remains unknown. In our vitro study, overexpression of miR-34a caused a significant decrease of *ATG9A* expression. Knockdown of *ATG9A* increased the expression of *p62* and blocked autophagosome–lysosome fusion, which is similar to the effect of miR-34a overexpression (Figure 7). These findings suggested that miR-34a modulation of autophagy at least partly through its target *ATG9A* in HEI-OC1 cells. It should be noted that miR-34a mediates autophagy via multiple targets, such as *SIRT1* and *Bcl-2*.^11, 52, 53, 54^ Future studies are undoubtedly needed to address the relationship between other targets of miR-34a and autophagy in HEI-OC1 cells.

Since increased miR-34a is correlated with AHL and cochlear hair cell loss in C57BL/6 mice,^11^ manipulation of miR-34a is proposed to be a potential intervention for prevention of AHL. As an effective miR-34a inhibitor, UDCA can be used as a cytoprotective agent to treat non-alcoholic fatty liver disease.^41^ Moreover, UDCA plays an antiapoptotic and anti-inflammatory role and is able to cross the blood–brain barrier; therefore, it was introduced as a novel approach for neurodegenerative diseases.^55, 56, 57^ However, the role of UDCA in autophagy modulation is controversial.^58, 59^ Our *in vitro* data showed that UDCA treatment decreased miR-34a levels and attenuated miR-34a-induced HEI-OC1 cell death. Also, UDCA alleviated *p62* changes induced by miR-34a overexpression, implying that UDCA rescued autophagic flux. These findings indicate that UDCA could protect HEI-OC1 cells from cell death by restoring the miR-34a-induced autophagic flux. Further studies are needed to confirm whether UDCA treatment is able to protect cochlear hair cells and delay AHL in the animal model.

## Conclusion

This study revealed that the impairment of autophagy and the elevation of miR-34a were found in the aging cochlea of C57BL/6 mice, which developed AHL. MiR-34a modulated autophagic flux via *ATG9A* and determined the fate of HEI-OC1 cells. Moreover, UDCA treatment prevented HEI-OC1 cells' death from restoring miR-34a-induced autophagy flux impairment. Our results increase our understanding of miR-34a-mediated autophagy in the control of cochlear hair cells fate and the development of AHL and propose miR-34a as a promising therapeutic target for the treatment of AHL.

## Materials and methods

### Animals

Sixty C57BL/6 mice (Laboratory Animal Center, Sun Yat-sen University, Guangzhou, China) were divided into three groups: 1 month old; 3 month old and 12 month old. Every group had 20 subjects. Animal care and experimental research were approved by the Animal Research Committee, Sun Yat-sen University and by the Animal Research: Reporting *In Vivo* Experiments guidelines.

### Auditory brainstem response

ABR measurements have been described in the previous study.^10^ The measurements were performed by inserting hypodermic needle electrodes at the vertex (active), below the left ear (reference), as well as the right ear (ground) after being narcotized by a peritoneal injection (100 mg/kg ketamine and 10 mg/kg xylazine mixture). The acoustic signals were generated, and using Tucker-Davis Technologies (TDT System III, Alachua, FL, USA) hardware and software processed the responses. Ten millisecond (ms) tone bursts with a 1 ms rise or fall time were presented at 4, 8, 16 and 32 kHz at a rate of 21.1/s. The average response to 1024 stimuli was gained through reducing the sound intensity at 5 dB intervals near the threshold. The lowest stimulation decibel level where a positive wave in the evoked-response trace was evident was defined and marked.

### Tissue preparation

The narcotized mice were killed after ABR recordings, and the cochleae were wiped off and fixed by steeping into 4% paraform in phosphate-buffered saline (PBS, pH 7.4) about 0.1 mM all night at 4 °C. After that, the cochleae were put into 4% sodium ethylenediaminetetraacetic acid for 2 days decalcification. For RNA and protein preparations, cochlear tissues were disassembled by minor tongs, quick frozen in liquid nitrogen and reserved at −80 °C.

### Surface preparations and hair cell counts

After 4% sodium ethylenediaminetetraacetic acid decalcification, the organ of Corti was microdissected, rinsed in precool PBS, immersed in 1% Triton X-100 for 15 min at room temperature, 100 *μ*l of phalloidin (Life Technology, Carlsbad, CA, USA) containing FITC at 37 °C in the dark for 120 min, followed by incubated with 4',6-diamidino-2-phenylindole) (10 mg/ml; Sigma, St. Louis, MO, USA) for 10 min, and mounted on glass slides in 50% glycerol. Samples were observed and imaged with an Olympus BX63 microscope (Olympus, Tokyo, Japan). We calculated two positions of the hair cell, at 10–20 and 65–70% of the whole cochlear duct distance from the apex, which approximately is in line with the 7–8 or 32–36 kHz frequency domain. Hair cells were calculated in six mice from each group and were considered to be degenerated if the cell nuclei were devoid.

### *In vitro* cell culture of HEI-OC1 cells

HEI-OC1 cells (kindly provided by F Kalinec at the House Ear Institute, Los Angeles, CA, USA) were cultured in Dulbecco's modified Eagle's medium (Gibco-Life Technologies, Carlsbad, CA, USA), supplemented with 10% fetal bovine serum (Gibco) at 33 °C under 10% CO_2_ (permissive conditions).

### Transfection of microRNA mimics, inhibitors and ATG9A siRNA

To examine the effect of miR-34a on autophagy and *ATG9A*, HEI-OC1 cells were transfected with a miR-34a mimic or a negative control of miRNA mimic (GenePharma, Shanghai, China) at 20 or 40 nM and harvested 24, 48 or 72 h later. In addition, HEI-OC1 cells were transfected with an *ATG9A* siRNA (Ruibo, Guangzhou, China) and harvested 72 h later to observe whether miR-34a regulate autophagy via *ATG9A*. The protective effect of UDCA (Sigma) was tested in HEI-OC1 cells under 10 *μ*M.

### Quantitative real-time polymerase chain reaction

According to the manufacturer's protocol, total RNA was isolated using TRIzol Reagent (Invitrogen-Life Technologies, Carlsbad, CA, USA), with 1 *μ*g of total RNA reverse-transcribed using a ReverTra-Plus-TM Kit (Takara, Osaka, Japan). Primer sequences used for amplifications were as follows: *LC3B* forward: 5′-TTATAGAGCGATACAAGGGGGAG-3′ and reverse: 5′-CGCCGTCTGATTATCTTGATGAG-3′ *p62* forward: 5′-GAACTCGCTATAAGTGCAGTGT-3′ and reverse: 5′-AGAGAAGCTATCAGAGAGGTGG-3′ glyceraldehyde-3-phosphate dehydrogenase: forward: 5′-TGAACGGGAAGCTCACTGG-3′ and reverse: 5′-GCTTCACCACCTTCTTGATGTC-3′. Complementary DNA samples were magnified using SYBR Premix Ex Taq (Takara) and detected with the Roche LightCycler 96 Real-Time PCR System (Roche, Basel, Switzerland). Glyceraldehyde-3-phosphate dehydrogenase was used to be internally piloting for *LC3B* and *p62* standardization. For miR-34a expressive assay, total RNA was isolated using TRIzol Reagent as well, with 500 ng of RNA reverse transcribed using special miRNA stem-loop primers and a PrimeScript RT reagent Kit (TaKaRa). According to the manufacturer's instructions,,mature miRNA expression was measured with TaKaRa Taq Version 2.0 plus dye (TaKaRa, Osaka, Japan) with miRNA levels normalized to U6 small nuclear RNA expression.

### Western blot analysis

Cultured cells and cochlear tissues were symmetrical on ice-cold radioimmunoprecipitation assay lysis buffer (Thermo Fisher Scientific, Pittsburgh, PA, USA) lasted about 30 min and centrifuged at 12 000 × *g* at 4 °C lasted 30 min, and the floating layer were gathered. Protein consistence was decided by using a protein test dye agentia (Bio-Rad, Hercules, CA, USA). Protein samples (20 *μ*g) were decomposed via sodium dodecyl sulfate polyacrylamide gel electrophoresis, shifting proteins onto a polyvinylidene fluoride membrane (Merck Millipore, Billerica, MA, USA) and blocked with 5% nonfat dry milk in TBS with 0.1% Tween 20 (TBS-T). The membranes were incubated with anti-*LC3B*, anti-*p62*, or anti-*ATG9A* (1 : 1000; Cell Signaling Technology, Danvers, MA, USA) overnight, washed three times (every 10 min) with TBS-T and incubated with a proper secondary antibody (1 : 10 000) for 1 h. After extensive membrane washing, the bands of immune reactivity were illustrated through Enhanced chemiluminescence (Merck Millipore). Band intensities were measured by densimetric test using NIH Image J (NIH, Bethesda, MD, USA), and *β*-actin was used as a loading and internally piloting to empower specimen standardization.

### Cell viability assay

Cell viability were examined by the MTS assay. HEI-OC1 cells in 100 *μ*l of nutrient medium were placed in 20 *μ*l of Cell Titer 96 Aqueous One Solution test (Promega, Madison, WI, USA) lasted 2 h according to the manufacturer's instructions and quantified at an absorbance of 490 nm using a Wellscan MK3 Microplate Reader (Labsystems Dragon, Helsinki, Finland).

### Transfection of cells with fluorescence LC3 adenoviral vectors

The mRFP-GFP-LC3 adenoviral vectors (Ad-mRFP-GFP-*LC3*) were provided by HanBio Technology Co. Ltd (HanBio, Shanghai, China). Dissection of the autophagic flux process by a novel reporter protein, tandem fluorescent-tagged LC3. The Ad was transfected into HEI-OC1 cells according to the manufacturer's protocol at an MOI of 100 for 6 h. Observation of autophagic flux was determined after fluorescent staining by evaluating the number of GFP and mRFP puncta (puncta/cell were counted).

### Statistical analysis

All experiments were independently repeated at least three times. Student's *t*-test and one-way analysis of variance with Fisher's *post hoc* test were used for statistical analysis. Values of *P*<0.05 were considered significant.
